# P-199. Implementation of a Diagnostic Stewardship Initiative for Hospital Onset *Clostridioides difficile* Infection

**DOI:** 10.1093/ofid/ofae631.403

**Published:** 2025-01-29

**Authors:** Caitlin Bettger, Bernadette Thompson, John L Kiley, Lisa Townsend

**Affiliations:** San Antonio Uniformed Services Health Education Consortium, Fort Sam Houston, Texas; Brooke Army Medical Center, San Antonio, Texas; BAMC, San Antonio, Texas; Brooke Army Medical Center, San Antonio, Texas

## Abstract

**Background:**

In response to increasing hospital onset CDI (HO-CDI), our Infection Prevention and Control (IPAC) Program undertook a diagnostic stewardship initiative with the goal of improving diagnostic accuracy of HO-CDI at our institution.

**Figure:**
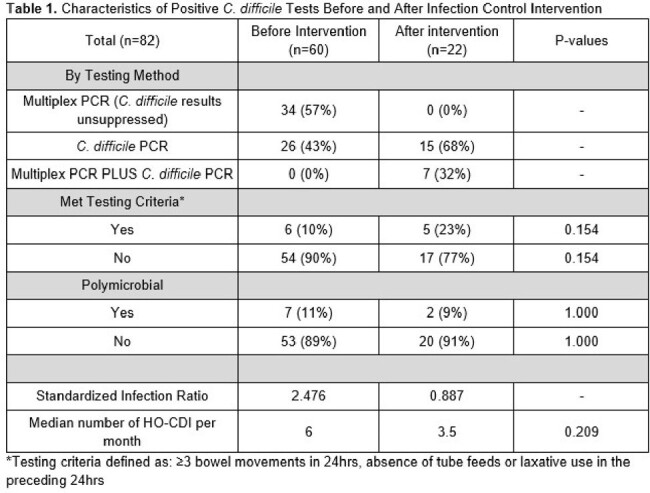

**Methods:**

Data acquisition was performed at a single center hospital with 425 inpatient beds as part of hospital surveillance. HO-CDI was defined by the National Health Surveillance Network (NHSN) definitions. A total of 18 months were examined (1/1/23 – 3/31/24). On 11/1/23, the IPAC Program in coordination with the Microbiology Laboratory began suppressing the results for C. difficile on the multiplex Gastrointestinal PCR with the goal of prompting providers to consider pre-test probability of diagnosis and order each PCR separately. Data was compared 10 months prior to implementation and 5 months after implementation. Groups were compared by Fisher exact for categorical variables and Mann-Whitney U for continuous variables.

**Figure:**
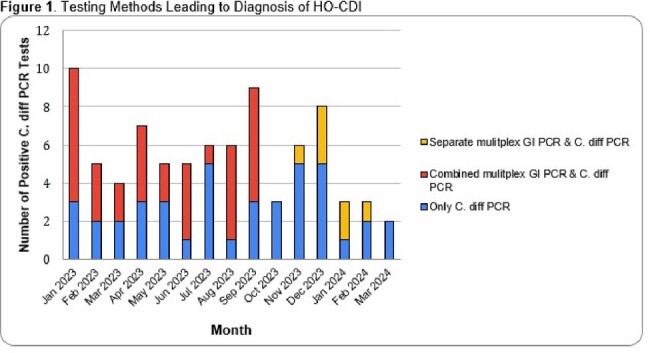

**Results:**

A total of 82 C. difficile PCR positive tests that met NHSN definitions for HO-CDI were recorded in the study period (Table 1). Of these, 13% of patients were deemed to have met testing criteria (≥3 bowel movements in a 24hr period, no tube feeds or laxatives in the preceding 24hrs). The most common potential disqualifier for testing was not meeting adequate number of bowel movements (47/82, 57%), followed by laxative use and tube feeds (44% and 28% respectively). Of all the positive tests, 9 (11%) were polymicrobial and none of the patients who had polymicrobial infections met testing criteria. The percentage of positive results coming from combined multiplex PCR and C. diff alone PCRs decreased after the intervention with 56% of positives being secondary to combined testing prior to the intervention compared to 32% after the intervention (p=0.078) (Fig 1). Median number of HO-CDI per month decreased after the intervention period along with the NSHSN standard infection rate (Fig 2).

**Figure:**
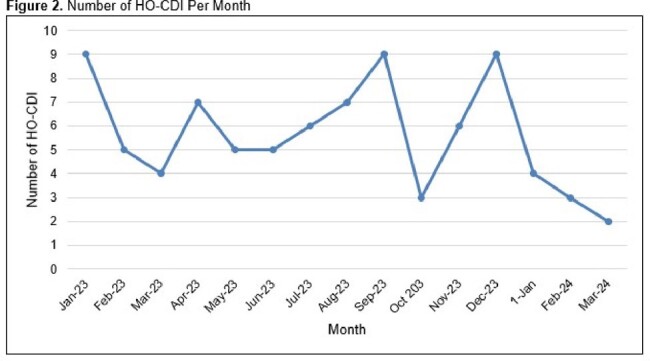

**Conclusion:**

Suppression of C. difficile results on multiplex Gastrointestinal PCR resulted in a decrease in this facility’s standard infection ration (SIR) for HO-CDI. Over three quarters of patients with positive results did not meet stool submission criteria. This has implications for possible next step educational initiatives.

**Disclosures:**

**All Authors**: No reported disclosures

